# White Matter Tract Segmentation as Multiple Linear Assignment Problems

**DOI:** 10.3389/fnins.2017.00754

**Published:** 2018-02-06

**Authors:** Nusrat Sharmin, Emanuele Olivetti, Paolo Avesani

**Affiliations:** ^1^NeuroInformatics Laboratory, Bruno Kessler Foundation, Trento, Italy; ^2^Center for Mind and Brain Sciences, University of Trento, Trento, Italy

**Keywords:** diffusion magnetic resonance imaging (dMRI), bundle/tract, tract segmentation, tractogram, nearest neighbor (NN), combinatorial optimization problem, linear assignment problem (LAP)

## Abstract

Diffusion magnetic resonance imaging (dMRI) allows to reconstruct the main pathways of axons within the white matter of the brain as a set of polylines, called streamlines. The set of streamlines of the whole brain is called the tractogram. Organizing tractograms into anatomically meaningful structures, called tracts, is known as the tract segmentation problem, with important applications to neurosurgical planning and tractometry. Automatic tract segmentation techniques can be unsupervised or supervised. A common criticism of unsupervised methods, like clustering, is that there is no guarantee to obtain anatomically meaningful tracts. In this work, we focus on supervised tract segmentation, which is driven by prior knowledge from anatomical atlases or from examples, i.e., segmented tracts from different subjects. We present a supervised tract segmentation method that segments a given tract of interest in the tractogram of a new subject using multiple examples as prior information. Our proposed tract segmentation method is based on the idea of streamline correspondence i.e., on finding corresponding streamlines across different tractograms. In the literature, streamline correspondence has been addressed with the nearest neighbor (NN) strategy. Differently, here we formulate the problem of streamline correspondence as a linear assignment problem (LAP), which is a cornerstone of combinatorial optimization. With respect to the NN, the LAP introduces a constraint of one-to-one correspondence between streamlines, that forces the correspondences to follow the local anatomical differences between the example and the target tract, neglected by the NN. In the proposed solution, we combined the Jonker-Volgenant algorithm (LAPJV) for solving the LAP together with an efficient way of computing the nearest neighbors of a streamline, which massively reduces the total amount of computations needed to segment a tract. Moreover, we propose a ranking strategy to merge correspondences coming from different examples. We validate the proposed method on tractograms generated from the human connectome project (HCP) dataset and compare the segmentations with the NN method and the ROI-based method. The results show that LAP-based segmentation is vastly more accurate than ROI-based segmentation and substantially more accurate than the NN strategy. We provide a Free/OpenSource implementation of the proposed method.

## 1. Introduction

The white matter of the brain mainly contains neuronal axons which are responsible for transferring signal between the regions of the gray matter (Douglas Fields, 2008). Diffusion magnetic resonance imaging (dMRI) (Basser et al., 1994) is the brain imaging technique for identifying the main pathways of large assemblies of axons and, in some cases, pathologies of the white matter (Douglas Fields, 2008). DMRI data quantify the local orientation of the axons of the white matter *in vivo*, at the voxel-level. From these local orientations, approximate 3*D* pathways can be reconstructed, using tractography algorithms. The resulting 3*D* polylines are called *streamlines* and the set of whole streamlines is called the *tractogram*, which usually contains a number of streamlines in the order of 10^5^–10^6^.

In neurological studies and neurosurgical planning, it is often important to identify the group of streamlines belonging to the same anatomical region, called *tract* or *bundle*, e.g., the corticospinal tract. The problem of grouping or organizing streamlines of the whole tractogram into anatomically meaningful tracts is known as the *tractogram segmentation problem* (O'Donnell and Westin, 2007). Moreover, extracting a specific tract of interest from the whole brain is known as the *tract segmentation problem* (Clayden et al., 2007). Once the segmentation is done, the statistical analysis over the tract(s) is used in multiple applications, for example, to study gender differences (Ingalhalikar et al., 2014), to observe the changes in age (Salat et al., 2005) and to correlate it with diseases (Bozzali et al., 2002; Park et al., 2004). This kind of studies require the analysis of groups of subjects. Therefore, automatic tract segmentation, conducted in a comparative manner over groups of subjects, is crucial in order to accomplish this goal. One main step of such task is to study how much *corresponding* are homologous anatomical structures across the subjects.

Several manual and automatic tract segmentation methods have been developed over the years. The manual approach, also known as *virtual dissection* (Catani et al., 2002; Mori et al., 2005; Wakana et al., 2007), extracts the desired tract using cortical regions of interest (ROIs) defined by neuroanatomical knowledge, by an expert. Such method is time consuming and it is limited to manual selection of ROIs. A different approach is the one of automatic tract segmentation, which can be divided into three different groups: *ROI-based*, also known as *parcellation-based, unsupervised* and *supervised*.

Automatic ROI-based tract segmentation is based on cortical parcellation. It requires that linear or non-linear registration is performed in advance, with respect to an anatomical atlas (Zhang et al., 2010; Aarnink et al., 2014). It has to be noted that the registration to the atlas can cause loss of information present in the diffusion images (Tunç et al., 2014). However, automatic ROI-based methods are known to provide remarkable results (Zhang et al., 2010) so, sometimes, they are used as ground truth to extensively validate the result of other segmentation methods. Among the parcellation-based automatic segmentation methods (Siless et al., 2016), the Tract Querier, based on the white matter query language (WMQL) (Wassermann et al., 2013, 2016), is one of the most adopted.

The unsupervised approach, usually called *fiber clustering*, is one of the most widely used tractogram segmentation technique in the literature (Shimony et al., 2002; Garyfallidis et al., 2012; Tunç et al., 2014; Reichenbach et al., 2015). The purpose of clustering is to group the streamlines according to their mutual geometrical similarity (or distance). Clustering methods mostly address single tractograms and not the joint analysis of multiple subjects (O'Donnell et al., 2013). A common criticism of clustering methods is that there is no guarantee to obtain anatomically meaningful tracts (Toga, 2015). For this reason, some authors propose to incorporate prior knowledge in the process, e.g., anatomical information from atlases or expert labeling on previous data.

Supervised segmentation is an approach based on prior knowledge. Prior knowledge can come from expert labeling of an anatomical atlas, see (Maddah et al., 2005), or from labeling clusters of streamlines from multiple subjects, in a process of atlas creation, see (O'Donnell and Westin, 2007; Guevara et al., 2012, 2017; Vercruysse et al., 2014; Yoo et al., 2015; Labra et al., 2017), or labeling clusters of streamlines from single subject in order to obtain segmented tract of interest (Garyfallidis et al., in press). In the former category, also known as *example-based tract segmentation*, once the atlas is available, the segmentation of the tract of interest in a new subject can be carried out. Existing example-based methods have some limitations, the main ones being the high computational cost (O'Donnell and Westin, 2007; Vercruysse et al., 2014), the dependency of the number of subjects to construct the atlas in case of multiple atlas (Guevara et al., 2012) and the dependency on the definition of threshold values (Yoo et al., 2015; Labra et al., 2017).

In supervised segmentation, besides atlases, other kinds of prior information can be used as prior knowledge, such as models or features of tracts/bundles. In this case, we call the task *model-based* supervised tract segmentation. Various parametric models of tracts have been proposed in the literature, e.g., the beta mixture model of similarity cosines (Clayden et al., 2007), the gamma mixture model of the distance (Maddah et al., 2008), the Gaussian process of tract probability map (Wassermann et al., 2010), the hierarchical Dirichlet process mixture model (Wang et al., 2011). Notice that models are built to describe tracts/bundles and not the whole tractogram.

In this work we propose an example-based tract segmentation, which segment a single specific tract from the whole tractogram of a new subject, using the prior information of the previous segmentations of the same tract/bundle from the tractogram of other subjects. The proposed method comprises two main steps: in the first step, for each streamline of an example tract, we find the corresponding streamline in the new tractogram. This step is related to our recent work in Sharmin et al. (2016), where we showed that streamline correspondence can be a powerful principle to transfer the anatomical information of a given bundle from one subject to another one. In the second step, we combine the correspondences found from all examples, i.e., from all other subjects, into one desired segmented tract within the new tractogram, using a ranking scheme. This second step accounts for the variability across the examples/subjects and the anatomical bias of the specificity of each subject. In the literature, given a set of streamlines of one subject, the problem of finding the corresponding streamlines in the tractogram of another subject has been addressed with a nearest neighbor strategy (Yoo et al., 2015; Labra et al., 2017; Garyfallidis et al., in press). There, after co-registering the two tractograms, for each streamline of the example subject, the corresponding streamline in the new tractogram has been proposed to be the geometrically nearest one. In this work, we compare the nearest neighbor strategy to that of finding corresponding streamlines as a Linear Assignment Problem (LAP, see Burkard et al., 2009), that we introduced in Sharmin et al. (2016). The LAP is a combinatorial optimization problem that finds corresponding objects between two sets imposing a one-to-one constraint, while minimizing the sum of the distances.

**Figure 1:**
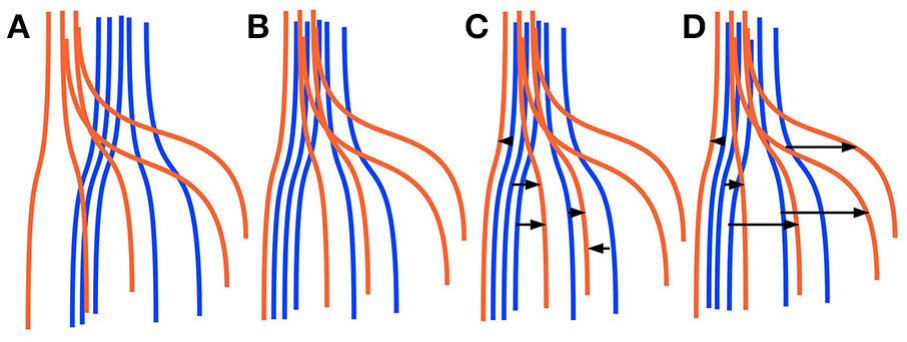
Simplified sketch of streamline correspondence showing the difference between nearest neighbor vs. the proposed linear assignment problem (LAP). In blue and red we show two sets of 5 homologous streamlines each, before registration **(A)**, after initial registration **(B)**, with arrows showing the correspondence from the blue streamlines to red ones with nearest neighbor (NN) **(C)** and with streamline correspondence with LAP **(D)**. NN **(C)** misses the two streamlines on the right side, while LAP **(D)** does not.

Finding corresponding streamlines through a LAP is different from using the nearest neighbor strategy. The main reason is that the nearest neighbor algorithm is greedy, i.e., it optimizes the selection of the correspondence individually for each streamline. Differently, the LAP jointly optimizes the correspondence of all streamlines, because of the one-to-one constraint. Intuitively, the benefit of this joint optimization can be seen in Figure 1, discussed below. In practical cases, it is common to observe some systematic displacement between homologous anatomical structures across subjects, even after an initial registration, because of the inherent variability of the local white matter anatomy across the population. In such cases, the nearest neighbor strategy is expected to provide poor correspondences: each streamline will be put in correspondence with its closest one in the other tractogram, without considering such systematic displacements. Differently, the one-to-one constraint of LAP will force the correspondence to follow such systematic displacements. We show a paradigmatic toy example of this difference in Figure 1. There, two sets of five simulated streamlines are depicted in blue and red before the initial alignment, see Figure 1A. Figure 1B illustrates the two sets of streamlines after linear registration. In Figures 1C,D we show the streamline correspondence between blue streamlines and red streamlines, through the nearest neighbor and the LAP strategies, respectively. There black arrows show the corresponding streamlines across the two sets. In Figure 1C, the nearest neighbor misses the two red streamlines on the right side. Differently, in Figure 1D, LAP correctly matches all the streamlines of the blue an red sets.

In this work, in addition to the main contribution of extending the correspondence-based segmentation of Sharmin et al. (2016) to the case of multiple tracts/bundles used as examples, we provide a substantial improvement in the speed of the computations, reducing the time required to segment, from hours to minutes. The newly adopted Jonker-Volgenant algorithm for LAP (LAPJV), see Bijsterbosch and Volgenant, 2010, for the rectangular LAP (RLAP), together with the use of an efficient way to compute the nearest neighbors of given streamlines, result in a 50x speed up of the computations with respect to what we presented in Sharmin et al. (2016). This improvement makes it possible to apply the proposed method in practical cases. Moreover, the efficient nearest neighbors computation can be used to vastly improve the speed of the method proposed in Yoo et al. (2015), similarly to Labra et al. (2017).

Together with the comparison against the nearest neighbors strategy, in this work, we compare the proposed method against the automatic ROI-based segmentation (Zhang et al., 2010), because it is the most adopted one. We conducted experiments on the dMRI data of multiple subjects from the Human Connectome Project (HCP, see Glasser et al., 2013; Sotiropoulos et al., 2013; Van Essen et al., 2013) dataset. We observed that the proposed LAP strategy, together with the proposed ranking scheme, are able to segment the bundles much more accurately than the ROI-based segmentation (Zhang et al., 2010) and the nearest neighbors strategy of Yoo et al. (2015). In the remaining part of the article, we first review the literature related to the different scientific areas touched by this work, see section 2. Then we introduce the necessary methodological ingredients, together with the detailed description of the proposed method, in section 3. The experiments are reported in section 5, followed by their discussion in section 6. We conclude this work by mentioning multiple future activities that arise from what we present.

## 2. Related works

### 2.1. Supervised tract segmentation

Here we review the literature on supervised tractogram segmentation and on the linear assignment problem. In order to organize the body of work in this field, we articulate the discussion on supervised tract segmentation along these five topics: alignment, embedding space, similarity/distance, correspondence techniques, and refinement step.

#### 2.1.1. Alignment

In supervised tract segmentation, tractograms are initially aligned to an atlas. Both voxel-based and streamline-based atlases have been used in literature, e.g., white matter ROI-based anatomical atlas (Maddah et al., 2005), high dimensional atlas (O'Donnell and Westin, 2007; Vercruysse et al., 2014), example-based single atlas (Guevara et al., 2012; Labra et al., 2017), example-based multi-atlas (Yoo et al., 2015). To the best of our knowledge, the specific step of alignment has been conducted with standard methods: in most of the cases with voxel-based linear registration (O'Donnell and Westin, 2007; Guevara et al., 2012; Yoo et al., 2015) and in the others with nonlinear voxel-based registration (Vercruysse et al., 2014).

#### 2.1.2. Embedding space

Streamlines are complex geometrical objects with a different number of points one from another. They are unfit to be directly given as input to many efficient data analysis algorithms which, instead, require vectors all with the same number of dimensions. Tractograms are large collections of streamlines, from hundreds of thousands to millions of streamlines, and their analysis is often limited by the required computational cost. A common preprocessing step before using algorithms like clustering, or nearest neighbor, is to transform streamlines into vectors, a process called *Euclidean embedding*. Different authors opted for different embedding approaches, like the spectral embedding (O'Donnell and Westin, 2007), the re-sampling of all streamlines to the same number of points (Guevara et al., 2012; Yoo et al., 2015; Labra et al., 2017), the use of B-splines with re-sampling (Maddah et al., 2005) and the dissimilarity representation (Olivetti and Avesani, 2011). Re-sampling all streamlines to a fixed number of points is the most common approach to obtain the embedding. In principle, up-sampling/down-sampling to a particular number of points may cause the loss of information. On the other hand, spectral embedding has high computation cost. The dissimilarity representation has shown remarkable results in terms of machine learning applications (Olivetti et al., 2013) and exploration of tractograms (Porro-Muñoz et al., 2015), at a moderate computational cost.

#### 2.1.3. Streamline distance

In order to find corresponding streamlines from one tractogram to another one, the definition of the streamline distance plays a crucial role. Most commonly, the corresponding streamline in the new tractogram is defined as the closest one, for the given streamline distance function. Similarly, when doing clustering for tract segmentation, the streamline distance function is a fundamental building block. Different streamline distance functions have been used in the supervised tract segmentation literature, e.g., minimum closest point (MCP) (O'Donnell and Westin, 2007), symmetric minimum average distance (MAM) (Olivetti and Avesani, 2011), minimum average flip distance (MDF) (Yoo et al., 2015; Garyfallidis et al., in press), Hausdorff distance (Maddah et al., 2005), normalized Euclidean distances (Labra et al., 2017), Mahalanobis distance (Yoo et al., 2015).

#### 2.1.4. Correspondence technique

One crucial aspect of supervised tract segmentation is the mechanism to find the corresponding streamline between the tractograms of different subjects, in order to transfer anatomical knowledge. A common approach for addressing such problem is to use the nearest neighbor strategy, i.e., finding the nearest streamline or centroid in the atlas and labeling the streamlines of the new subject based on that. In O'Donnell and Westin (2007) and O'Donnell et al. (2017), a high dimensional atlas was reconstructed from multiple tractograms. Then the new subject was aligned with the atlas and the closest cluster centroids from atlas were computed to assign the anatomical label. In Guevara et al. (2012), nearest centroids of the new subject were computed from a single atlas from multiple subjects, with the normalized Euclidean distance. Recently, a faster implementation has been proposed in Labra et al. (2017). There, they proposed to label each single streamline instead of cluster centroids and to accelerate the computation time by filtering the streamlines in advance, using properties of the normalized Euclidean distance. A limitation is that an appropriate threshold has to be defined for each tract to be segmented. Similarly, in Yoo et al. (2015), the nearest neighbor strategy is used to find corresponding streamlines between those of the tractogram of a new subject, with those of multiple example-subjects (12, in their experiments). Again, two thresholds, i.e., a distance threshold and a voting threshold, are required to be set in order to obtain the segmentation. The proposed implementation requires a GPU.

A different approach based on graph matching, instead of the nearest neighbor, was proposed by us for tractogram alignment, see Olivetti et al. (2016). Such idea could be extended to the tract segmentation problem.

#### 2.1.5. Refinement

After segmentation, in order to improve the accuracy of the segmented tract, some authors propose a refinement step, for example, to identify and remove outliers. In Mayer et al. (2011), a tree-based refinement step was introduced. Initially, they padded the segmented tract with the nearest neighbors and then used a probabilistic boosting tree classifier to identify the outliers. Another approach to increase the accuracy of the segmented tract is majority voting (Rohlfing et al., 2004; Jin et al., 2012; Vercruysse et al., 2014; Yoo et al., 2015). The main concept of the majority voting is to reach the agreement on the segmented streamlines (or voxel) coming from different examples, usually removing the infrequent ones.

The accuracy of the outcome after the step of refinement is closely related to the number of examples. This relation has been investigated in the vast literature of multi-atlas segmentation (MAS). The intuitive idea is that the behavior of the segmentation error is connected to the size of the atlas dataset. A first attempt to characterize such a relationship, with a first principle approach, was proposed by Awate and Whitaker (2014). In their proposal the size of the atlases is predicted against the segmentation error by formulating the multi-atlas segmentation as a nonparametric regression problem. More recently, Zhuang and Shen (2016) combined the idea of multi-atlas with multi-modality and multi-scale patch for heart segmentation. For a comprehensive survey of multi-atlas segmentation in the broader field of medical imaging, see Iglesias and Sabuncu (2015).

### 2.2. Linear assignment problem solutions

The linear assignment problem (LAP) computes the optimal one-to-one assignment between the *N* elements of two sets of objects, minimizing the total cost. The LAP takes as input the cost matrix that describes the cost of assigning each object of the first set to each object of the second set. Various algorithms for solving the LAP in polynomial time has been proposed in literature. A comprehensive review of all the proposed algorithms can be found in Burkard et al. (2009) and Burkard and Cela (1999). An extensive computational comparison among eight well known algorithms is in Dell'Amico and Toth (2000). The algorithms are: Hungarian, signature, auction, pseudoflow, interior point method, Jonker-Volgenant (LAPJV). According that survey and to Serratosa (2015), the Hungarian (Kuhn, 1955) algorithm and Jonker-Volgenant algorithm (LAPJV, Jonker and Volgenant, 1987) are the most efficient ones, with time complexity $O\left(N^{3}\right)$. Nevertheless, in practice, LAPJV is much faster than the Hungarian algorithm, as reported in Serratosa (2015) and Dell'Amico and Toth (2000). This occurs because, despite the same time complexity class, i.e., $O\left(N^{3}\right)$, the respective constants of the 3rd order polynomials describing the exact running time of each algorithm are much different, giving large advantage to LAPJV. We have directly observed this behavior in our experiments with LAPJV, compared to those with the Hungarian algorithm that we published in Sharmin et al. (2016).

According to Dell'Amico and Toth (2000) and Burkard et al. (2009), LAPJV is faster than other algorithms in multiple applications, see also Bijsterbosch and Volgenant (2010). Moreover, in many practical applications, the two sets of objects^1^ on which to compute the LAP have different sizes, i.e., the related cost matrix is rectangular. In Bijsterbosch and Volgenant (2010), the rectangular version of LAPJV was proposed with a more efficient and robust solution than the original one in Jonker and Volgenant (1987). In this work, we adopted the rectangular version of LAPJV because of its efficiency and because we need to compute the correspondence between an example tract and the target tractogram which, clearly, have different number of streamlines.

## 3. Methods

In this section, we first introduce basic definitions, terminology and some basic computational tools used in the proposed method. Afterwards, we provide all details of the proposed tract segmentation method. The proposed method provides automatic segmentation of a tract/bundle of interest from a set of examples from different subjects. The proposed method comprises two main steps: in the first, a preliminary segmentation of the tract is obtained from each example. This step is accomplished by casting the segmentation problem as a rectangular linear assignment problem (LAP), which we implement with the rectangular version of Jonker-Volgenant algorithm (LAPJV). The second step merges the segmentations coming from different examples, with a ranking scheme. In this way, it is possible to obtain the final segmentation of the tract of interest that comprises the anatomical information coming from the different examples/subjects.

### 3.1. Basic definitions and tools

Here we provide basic definitions and the description of some computational tools used in the proposed method, namely: (i) a distance function between streamlines, (ii) the dissimilarity representation, that we use as Euclidean embedding for streamlines and (iii) the *k*-d tree data structure, for fast nearest neighbor queries. We also formally define the linear assignment problem.

A *streamline s* is a polyline in 3*D* space, i.e., *s* = {**x**_1_, **x**_2_, …**x**_*n*_*s*__}, where ${\text{x}}_{i}\in {\mathbb{R}}^{3}$. Let *T* = {*s*_1_, *s*_2_, …, *s*_*M*_} be the whole brain *tractogram*, represented as a set of *M* streamlines^2^. A *tract*, also called *bundle*, is denoted as *t* ⊂ *T*. Note that, in this work, *tract* will be referred as a set of streamlines with particular anatomical meaning, e.g., the cortico-spinal tract (*CST*).

#### 3.1.1. Streamline distance

In the literature, multiple inter-streamline distance functions have been proposed (Garyfallidis et al., 2012). In this work, we adopt the most common one, i.e., the symmetric minimum average mean distance (MAM) (Garyfallidis et al., 2012). Given two streamlines *s*_*a*_ and *s*_*b*_:

(1) $$\begin{array}{l} d_{MAM}\left(s_{a},s_{b}\right)=\frac{1}{2}\left(D\left(s_{a},s_{b}\right)+D\left(s_{b},s_{a}\right)\right) \end{array}$$

where $D\left(s_{a},s_{b}\right)=\frac{1}{\left|s_{a}\right|}\overset{\left|s_{a}\right|}{\underset{i=1}{\sum }}d\left({\text{x}}_{i}^{a},s_{b}\right)$ and $d(x_{i}^{a},s_{b})=\min_{j=1,\ldots ,|s_{b}|}||x_{i}^{a}-x_{j}^{\prime }|{|}_{2}$.

#### 3.1.2. Dissimilarity representation

The *dissimilarity representation* (DR) is a Euclidean embedding technique to represent generic objects in a vector space (Pekalska and Duin, 2005) when a distance between the objects is available. In this work, we use the DR to represent the streamlines in vector space^3^, as proposed in Olivetti et al. (2012). The DR is defined by the distance function between streamlines and by defining a (usually small) subset of the tractogram, called the *prototypes*. Given the distance and the prototypes, each streamline is then represented as the vector of the distances from the prototypes. Given a tractogram *T* and the set of prototypes $\Pi =\left\{s_{1}^{*},s_{2}^{*}\ldots ,s_{p}^{*}\right\}$, the dissimilarity representation of *s* ∈ *T* is the *p*-dimensional vector:

(2) $$\begin{array}{l} x_{\Pi }\left(s\right)=\left[d\left(s,s_{1}^{*}\right),d\left(s,s_{2}^{*}\right),\ldots ,d\left(s,s_{p}^{*}\right)\right]. \end{array}$$

As shown in Olivetti et al. (2012) and Porro-Muñoz et al. (2015), the DR of streamlines is very accurate with a few tens of prototypes selected by means of the Subset Farthest First (SFF) algorithm, which requires just a few seconds of computation.

#### 3.1.3. *K*-d (*k*-dimensional) tree

A *k*-dimensional tree (*k*-d tree, see Bentley, 1975) is a space partitioning data structure to efficiently store and retrieve vectorial data in k-dimensional space. The *k*-d tree provides fast nearest neighbor queries on large datasets. In this work, we use the *k*-d tree on the vectorial representation of streamlines, in order to quickly find the neighboring streamlines of a given streamline *s*. The computational time complexity of building a *k*-d tree is O(*M*log*M*) and the nearest neighbor query has time complexity O(log*M*), where *M* is the number of streamlines of the tractogram. For example, a modern desktop computer can retrieve the 10 neighboring streamlines of a given streamline from a whole tractogram in just a few milliseconds^4^.

#### 3.1.4. Linear assignment problem

The Linear Assignment Problem (LAP) is a fundamental combinatorial optimization problem. Given two sets of objects *A* = {*a*_1_, …, *a*_*M*_} and *B* = {*b*_1_, …, *b*_*M*_} and the cost matrix $C={\left\{c_{ij}\right\}}_{ij}\in {\mathbb{R}}^{M\times M}$ that provides the cost *c*_*ij*_ of assigning/matching *a*_*i*_ to *b*_*j*_, the LAP aims to find the *one-to-one* correspondence between the two sets that has the minimum total cost. In our context, we use the LAP to find the correspondence between two sets of streamlines, as we recently proposed in Sharmin et al. (2016). In our case, the cost to minimize is the distance between the streamlines, i.e., $c_{i,j}=d\left(s_{i}^{A},s_{j}^{B}\right)$. A one-to-one assignment can be represented with a binary matrix *P* = {_*p*_*ij*_}*ij*_, where *p*_*ij*_ = 1 if *a*_*i*_ corresponds to *b*_*j*_ and zero otherwise. The one-to-one constraint makes *P* a *permutation matrix*, i.e., only one element in each row and column is 1. Then, the LAP is defined as

(3) $$\begin{array}{l} P^{*}=\underset{P\in P}{\text{argmin}}\overset{M}{\underset{i,j=1}{\sum }}C_{ij}p_{ij}. \end{array}$$

where P^*^ is the optimal assignment and P is the space of all possible assignments. When the size of the two sets differs, i.e., |*A*| ≠ |*B*|, the problem is called *rectangular* linear assignment problem (RLAP) (Bijsterbosch and Volgenant, 2010), a generalization of the LAP. Further information is provided below, in section 3.2.1.

### 3.2. Proposed tract segmentation framework

**Figure 2:**
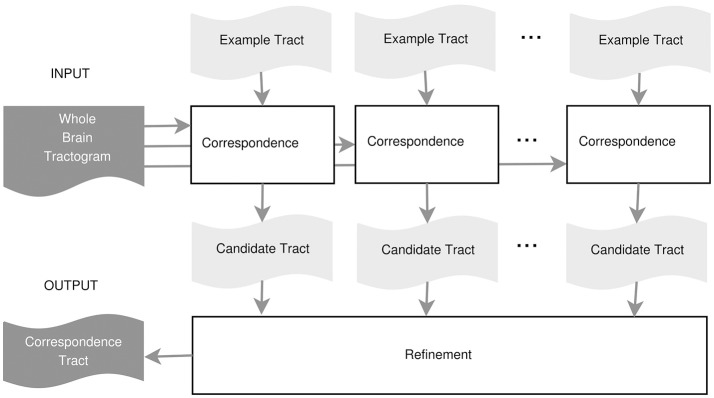
Proposed tract segmentation framework.

**Figure 3:**
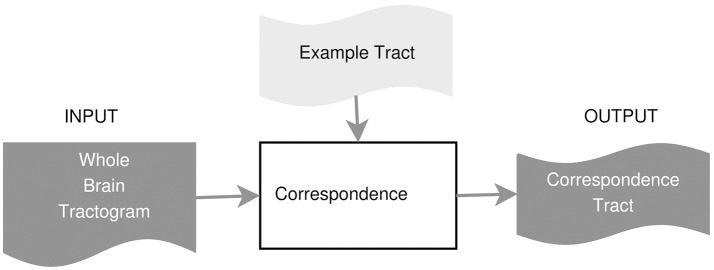
Tract segmentation from a single example.

In this work, we propose an example-based supervised tract segmentation approach. Given an anatomical tract of interest, e.g., the corticospinal tract (CST), and a set of examples of that tract, segmented from multiple subjects, our aim is to exploit the information from those examples in order to automatically segment the tract of interest in a new subject. The tract segmentation method that we propose consists of two main steps, illustrated in Figure 2. In the first step, each example tract is used to obtain a candidate segmentation in the new tractogram, see Figure 3. In the second step, the candidates generated from each example are merged together and then filtered, in order to obtain the final segmentation of the desired tract, taking into account the variability of the examples across subjects. In the following, we describe the details of the two main steps in section 3.2.1 and section 3.2.2, respectively.

#### 3.2.1. Step 1: tract segmentation from a single example

In this part, we describe the details of how to compute the corresponding streamlines of a single example tract, using the either a fast nearest neighbor algorithm or the linear assignment problem. Given one example of the tract of interest, $t_{A}=\left\{s_{1}^{A},\ldots ,s_{k}^{A}\right\}$, e.g., the segmented CST from subject *A*, the first step of the proposed method is able to extract the set of streamlines of subject *B* corresponding to *t*_*A*_, that we call *t*_*A*→*B*_ ⊂ *T*_*B*_. The extracted tract, *t*_*A*↦*B*_, is an approximation of the actual tract/bundle of interest in subject *B*, i.e., *t*_*B*_. The procedure is based on the concept of streamline correspondence, i.e., on finding which streamline in the new tractogram corresponds to a given streamline in the example tract.

##### Correspondence as nearest neighbor

As explained in section 2, the literature reports that the most common strategy to obtain corresponding streamlines across two subjects is based on the idea of Nearest Neighbor (NN). Assuming that the set of streamlines of two subjects are registered in a common space, usually by means of an affine transformation, the streamline of the subject *B* corresponding to a given $s_{i}^{A}$ of subject *A*, is defined as the closest (nearest) one

(4) $$\begin{array}{l} s_{A,i}^{B}=\underset{s^{B}\in T_{B}}{\text{argmin}}\;d\left(s^{B},s_{i}^{A}\right) \end{array}$$

In principle, the task of retrieving neighboring streamlines has high computational cost. The straightforward solution entails computing the distance from the given streamline to all other streamlines in the new/target tractogram and then getting the one with minimum distance. The approaches currently adopted in the literature are more efficient and based on several pre-processing steps, that may involve clustering, the definition of thresholds and the use of fast graphics processing units (GPUs), see Yoo et al. (2015) and Labra et al. (2017). In this work, we claim that segmentation based on nearest neighbor is fairly sub-optimal and can be improved with the idea of linear assignment (see below). Nevertheless, in this work we also propose the following procedure to greatly simplify such computation of nearest neighbors, both in terms of logical steps and time:

1. Compute a vectorial representation, e.g., the dissimilarity representation (DR), of the streamlines of *T*_*B*_ and of *t*_*A*_^5^.
2. Build a *k*-d tree from the vectors of *T*_*B*_.
3. For each streamline in *t*_*A*_, in vector form, perform a nearest neighbor query on the *k*-d tree and obtain the closest streamline.

##### Correspondence as (rectangular) linear assignment problem

The segmentation method that we propose in this work is based on the (rectangular) linear assignment problem (LAP). We observed that finding corresponding streamlines with the nearest neighbor strategy is suboptimal. A typical problem with the nearest neighbor is that homologous anatomical structures of two different subjects may have a systematic displacement even after the initial affine registration. In those cases, the nearest neighbor strategy would be too greedy and would fail to retrieve the correct corresponding streamlines because of the systematic displacement, selecting instead the closest ones, see Figure 1. For this reason, we claim that introducing the further constraint of *one-to-one* correspondence between streamlines forces the correspondence to follow the local displacements that may occur.

Given the tract/bundle $t_{A}=\left\{s_{1}^{A},\ldots ,s_{k}^{A}\right\}$ of subject *A* and the tractogram $T_{B}=\left\{s_{1}^{B},\ldots ,s_{M}^{B}\right\}$ of subject *B*, where *k* ⋘ *M*, and a distance function *d*() between streamlines, we denote as *C* = [_*c*_*ij*_]*i* = 1…*k, j* = 1…*M*_, the *k* × *M* distance matrix between each streamline of *t*_*A*_ and each of *T*_*B*_, i.e., $c_{ij}=d\left(s_{i}^{A},s_{j}^{B}\right)$. Then, we define the corresponding streamlines of *t*_*A*_ in *T*_*B*_ as those found by the solution of the corresponding rectangular linear assignment problem (RLAP):

(5) $$\begin{array}{l} P^{*}=\underset{P\in P}{\text{argmin}}\overset{k}{\underset{i=1}{\sum }}\overset{M}{\underset{j=1}{\sum }}c_{ij}p_{ij} \end{array}$$

where ${\left[p_{ij}\right]}_{ij}=P\in P$ is a *partial* permutation matrix, i.e., $P={\left[p_{ij}\right]}_{ij}\in {\left\{0,1\right\}}^{k\times M}$ and $\overset{k}{\underset{j=1}{\sum }}p_{ij}=1$ but $\overset{M}{\underset{i=1}{\sum }}p_{ij}\leq 1$, because the tract has less streamlines then the tractogram. P^*^ is the optimal assignment, i.e., the one with lowest overall cost. Once P^*^ is obtained, the segmented tract in *T*_*B*_ is defined as the set streamlines in *T*_*B*_ corresponding to *t*_*A*_ according to P^*^.

##### Solution of the linear assignment problem

Finding the optimal solution of the LAP or RLAP is usually computationally very expensive. As mentioned before, we adopted the rectangular LAPJV that we do not describe here because its description is fairly technical. We refer the interested reader to Bijsterbosch and Volgenant (2010) and Jonker and Volgenant (1987) for a comprehensive description, as to section 2 for a concise analysis of the literature on this topic. Anyway, a key element of the scalability of our solution, is a further step that we introduce before starting the RLAP: the *sparsification* of the cost matrix *C*. In our experiments, we noticed that only a small part of the whole *C* is actually used in LAPJV. Specifically, for each streamline in *t*_*A*_ only the distance from the neighboring streamlines in *T*_*B*_ play a role in the computation. For this reason, by leveraging the DR and the *k*-d tree introduced above, we efficiently computed just a small subset of *C* instead of the whole matrix, saving a substantial amount of time and memory both in building *C* and in executing LAPJV. In section 5, we show the details of this step and the gain obtained.

#### 3.2.2. Step 2: tract correspondence from multiple examples

In this part we describe how to combine multiple solutins of tract correspondence from one example tract into a single segmented tract on the target tractogram. The quality of the segmented tract/bundle obtained from a single example, as described in section 3.2.1, is limited by inherent differences in the white matter anatomy between subject *A* and *B*. In order to reduce the bias of segmenting from a single specific example, here we describe a procedure to segment the desired tract/bundle from multiple examples, each from a different subject. In this way we try to exploit the anatomical variability in the sample of subject as if we had a model of the tract. The proposed procedure is based on first computing the correspondence between each example and the new tractogram, as detailed in section 3.2.1, and then on ranking all the corresponding streamlines based on how frequently they were selected by the segmentation induced by each example. In the following, we describe the details of the proposed procedure.

##### Refinement step: ranking schema

Given a tract/bundle of interest *t*, e.g., the CST, the set of *N* examples segmentations of that tract from *N* different subjects is denoted as {*t*_*A*_1__, …, *t*_*A*_*N*__}. For each example tract *t*_*A*_*i*__, a corresponding tract *t*_*A*_*i*_→*B*_ ⊂ *T*_*B*_ can be obtained following the RLAP procedure explained in section 3.2.1, or other strategies like the nearest neighbor. The resulting (approximate) segmentations for *t*_*B*_ are then {*t*_*A*_1_→*B*_, …, *t*_*A*_*N*_→*B*_}. Our aim is to find an improved approximation of *t*_*B*_, that we call ${\hat{t}}_{B}$, leveraging all of them. The proposed procedure to obtain ${\hat{t}}_{B}$ is the following:

1. Compute the union of the obtained segmentations: $t_{\left(A_{1},\ldots ,A_{N}\right)\to B}={⋃}_{i=1}^{N}t_{A_{i}\to B}$, which we expect to be a superset of *t*_*B*_.
2. Score each streamline in *t*_(_*A*__1_, …, *A*_*N*_) → *B*_ ⊂ *T*_*B*_ by counting how many time the streamline appears in {*t*_*A*_1__, …, *t*_*A*_*N*__}. In this way, being selected multiple times as potential streamline of *t*_*B*_ is considered evidence of being a reliable streamline. We further introduce other criteria in order to break ties in the ranking between streamlines selected the same number of times. Our proposed second criterion comes from the selection cost of the streamlines, i.e., the distance of the streamline during the time of selection. Lower cost means a more reliable matching. Once all scores are obtained, we ranked the streamlines according to their score.
3. We define the number of streamlines of ${\hat{t}}_{B}$ as the median number of streamlines of the example tracts: $\hat{k}=|{\hat{t}}_{B}|=median\left(t_{A_{1}\to B},\ldots ,t_{A_{N}\to B}\right)$.
4. ${\hat{t}}_{B}$ is the set of $\hat{k}$ top scoring streamlines.

## 4. Materials

In this section, we describe the datasets used to conduct the experiments, as well as the procedure to obtain to ground truth of the segmentations, i.e., through the white matter query language (WMQL). A total amount of 300 segmented tracts will be considered in our experiments.

### 4.1. Datasets

Thirty healthy subjects from the Human Connectome Project (HCP) (Sotiropoulos et al., 2013) public datasets were selected at random and their MRI data retrieved. The diffusion MRI dataset from HCP consists of 3 × 90 gradient directions at *b*-values of 1,000, 2,000, and 3,000 s/mm^2^, with 18 non-diffusion weighted images, with voxel size of 1.25 mm. The fiber orientation distribution (FOD) function at each voxel was computed using the constrained spherical deconvolution algorithm (CSD) (Tournier et al., 2007) with a single shell (*b* = 1,000). The tractograms were obtained with the Euler Delta Crossing (EuDX) algorithm, see (Garyfallidis et al., 2014), implemented in DiPy^6^, with 10^6^ seeds. The number of streamlines of the resulting tractograms is approximately 100–140 thousands.

### 4.2. Tractogram segmentation with WMQL

The TractQuerier, based on the White Matter Query Language (WMQL) (Wassermann et al., 2013, 2016), is a parcellation-based Open Source tool^7^ to segment the whole brain tractogram. Using the parcellation from FreeSurfer and a database of ROIs, defined by the anatomical information of the tract, the WMQL extracts the anatomically meaningful tracts with a rule-based approach. Here, WMQL is applied over thirty subjects from the HCP dataset to produce a set of tracts, specifically: corticospinal tract (CST), cingulum (CG), arcuate fasciculus (AF), inferior longitudinal fasciculus (ILF), Uncinate Fascicle(UF), inferior fronto-occipital fasciculus (IFOF), 1, 2, and 3 left-right superior longitudinal fasciculus (SLF1,2,3), and finally, corpus callosum (CC) with seven parts (CC1-7). Among all the tracts, we selected those showing more consistency across subjects, resulting in a set of ten different tracts: left and right corticospinal tract (CST), left and right cingulum (CG), left and right inferior fronto-occipital fasciculus (IFOF), left and right Uncinate Fascicle (UF) and left and right inferior longitudinal fasciculus (ILF). These tracts will be used as *exampled tracts*, as well as for the quantitative comparison of the tract segmentation methods. Note that no further manual filtering is performed on the segmented tracts. In Table 1, we report the mean and standard deviation of the number of streamlines and of voxels over 30 subjects for the ten selected tracts.

**Table 1:** Data description.

**Tract name**	**No. of streamline**	**No. of voxels**
CST.Left	39 ± 39	957 ± 625
CST.Right	23 ± 34	644 ± 544
CG.Left	1, 141 ± 168	9, 039 ± 1, 172
CG.Right	982 ± 159	8, 200 ± 1, 087
IFOF.Left	173 ± 94	3, 713 ± 1, 295
IFOF.Right	123 ± 76	2, 796 ± 1, 212
ILF.Left	96 ± 71	1, 771 ± 610
ILF.Right	54 ± 59	2, 035 ± 566
UF.Left	143 ± 76	1, 794 ± 844
UF.Right	185 ± 66	1, 140 ± 819

*For each tract, the table reports the mean and standard deviation of the number of streamlines and of voxels over 30 subjects*.

## 5. Experiments

In this section, we present all the components for the quantitative validation of our proposed supervised tract segmentation method based on LAP. Experiments were performed on 30 subjects from the Human Connectome Project (HCP) dMRI dataset, as described in section 4. The tractograms of all subjects were aligned to the MNI152 template with the voxel-based linear registration tool FLIRT/FSL^8^. After alignment, the WMQL was applied over thirty subjects to segment the whole brain tractogram and obtain 10 different tracts: left-right corticospinal tract (CST), left-right cingulum (CG), left-right inferior fronto-occipital fasciculus (IFOF), left-right Uncinate Fascicle (UF) and left-right inferior longitudinal fasciculus (ILF). We divided the set of subjects into two groups, i.e., the *example group* and the *test group*, each of 15 subjects. The tracts from the example group were used as prior information to guide the segmentation in the tractogram of new subjects. The tracts from the test group were used to quantify the quality of the obtained segmentation.

**Figure 4:**
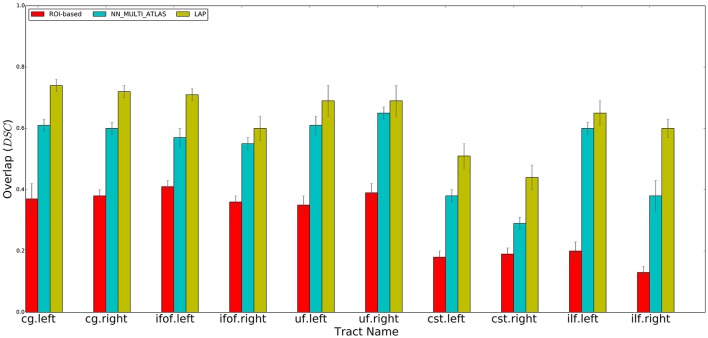
Summary of the results: average voxel overlap between segmented tracts and ground truth tracts for the ROI-based, NN_MULTI_ATLAS, and the proposed LAP. The bar graph shows the degree of overlap, in terms of dice similarity coefficient (DSC), for each tract averaged over 15 subjects.

**Figure 5:**
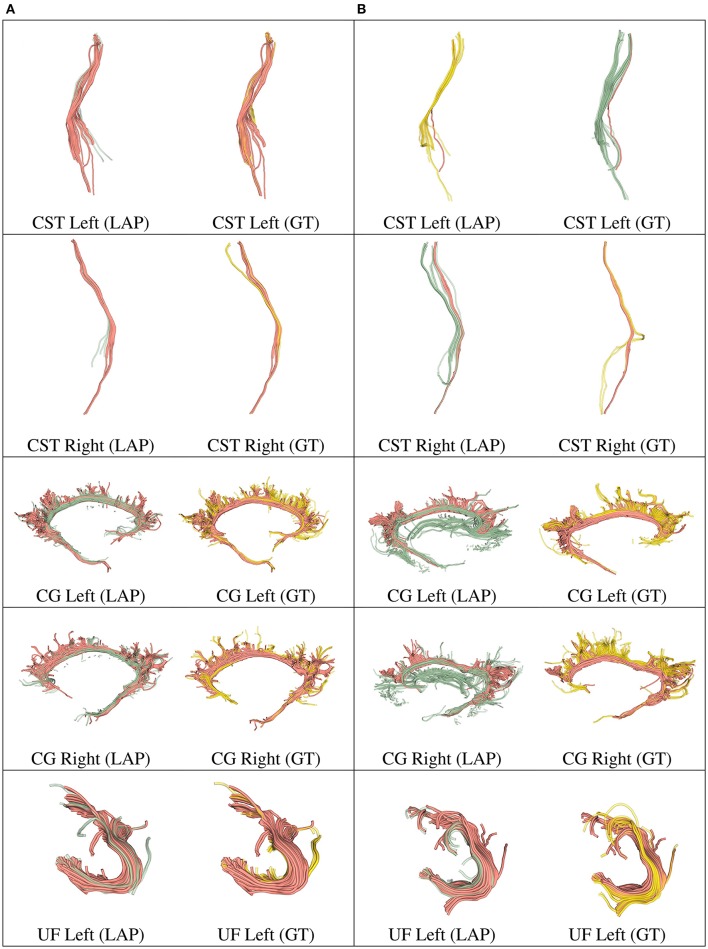
In each row, in column **(A)**, we show the tract obtained with LAP-based segmentation (LAP) with highest DSC score among all test subjects, together with the related ground truth (GT). In column **(B)**, we show the one with lowest DSC score, together with the ground truth. The streamline of salmon color are TP, the ones in green are FP and the ones in yellow are FN. The tracts are: CST (left, right), CG (left, right) and UF (left).

**Figure 6:**
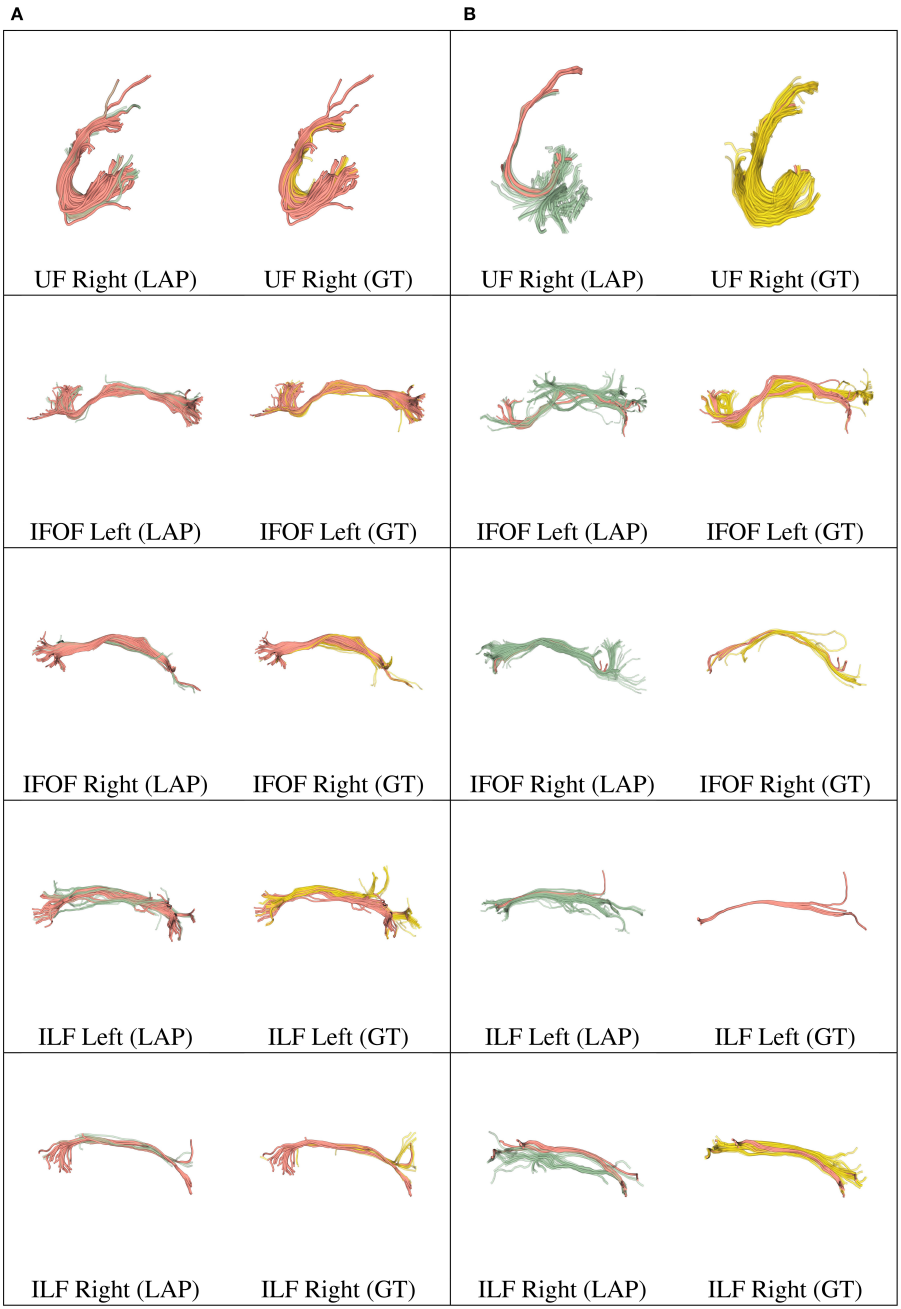
In each row, in column **(A)**, we show the tract obtained with LAP-based segmentation (LAP) with highest DSC score among all test subjects, together with the related ground truth (GT). In column **(B)**, we show the one with lowest DSC score, together with the ground truth. The streamline of salmon color are TP, the ones in green are FP and the ones in yellow are FN. The tracts are: and UF (right), IFOF (left, right) and ILF (left, right).

**Figure 7:**
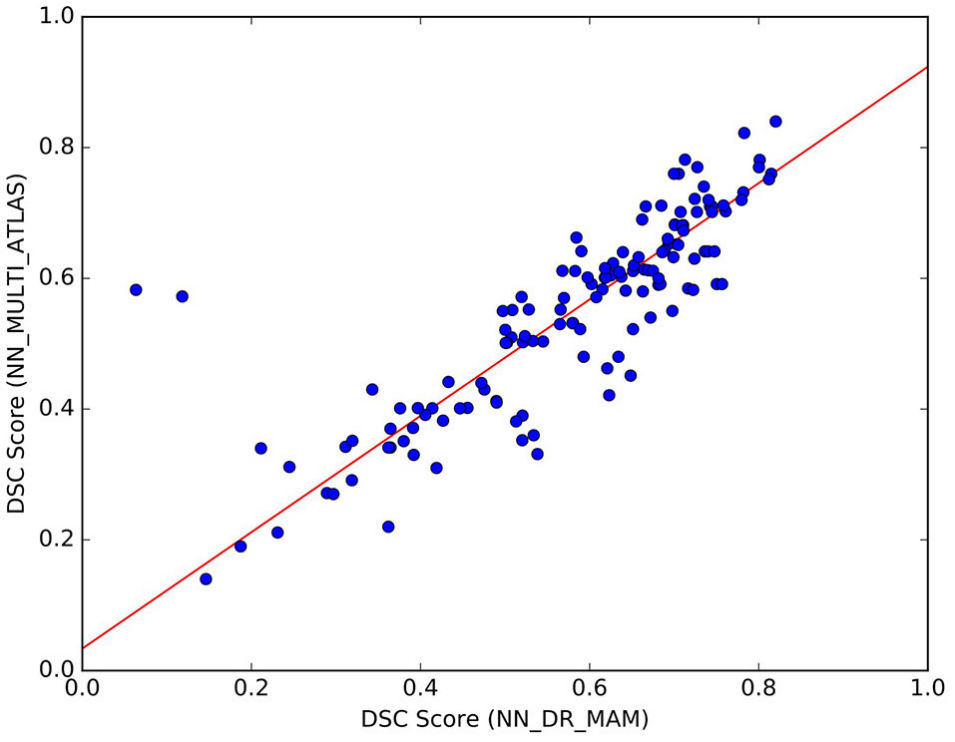
Comparison of the results between NN_DR_MAM and NN_MULTI_ATLAS. For each of the 10 tracts of the 15 test subjects (10 × 15) = 150 points in total), the graph reports DSC computed with NN_DR_MAM (x-axis) and the NN_MULTI_ATLAS method (y-axis).

**Figure 8:**
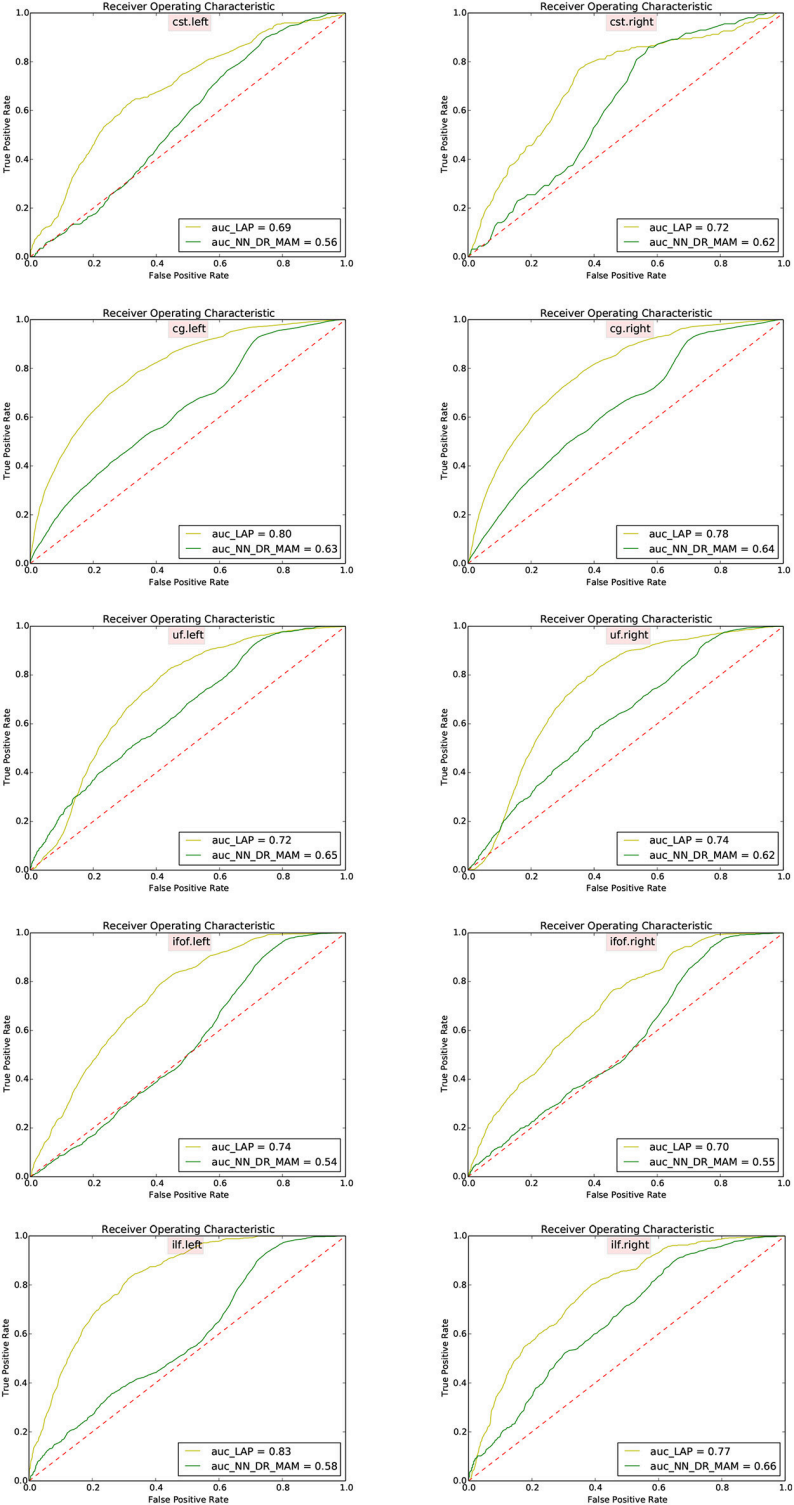
Receiver operating characteristic (ROC) analysis between segmented tracts and ground truth tracts for the NN_DR_MAM and the proposed LAP. Each subfigure shows the average ROC curve for each tract averaged over 15 subjects. For each tract the voxel information has consider for the ROC curve analysis and For each subject different thresholds has chosen.

**Figure 9:**
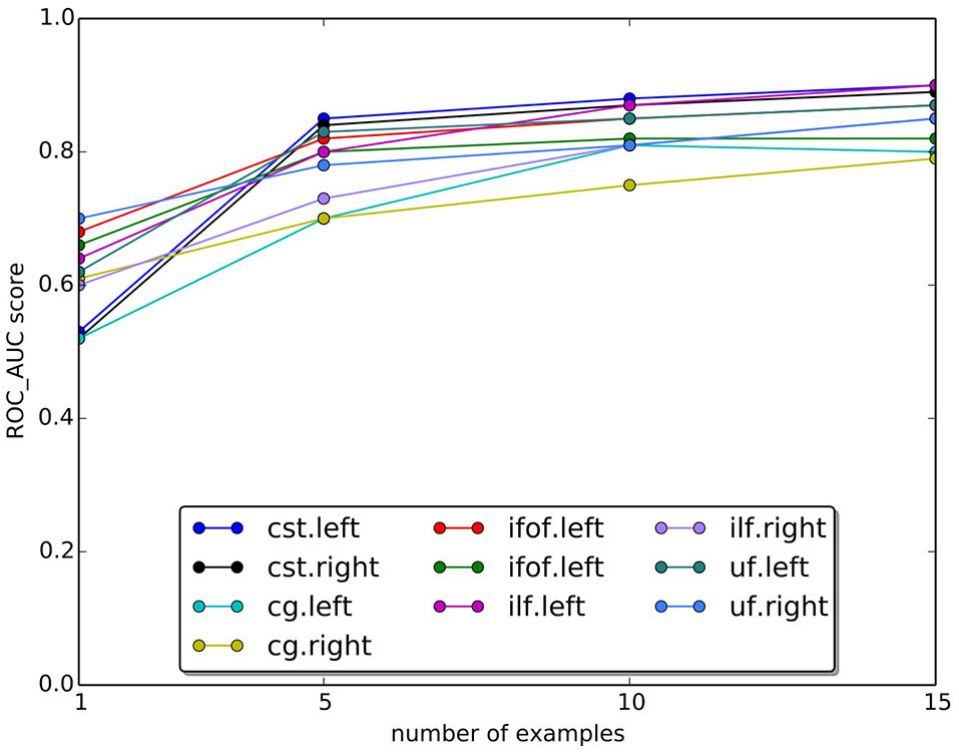
Change in the AUC score with the number of examples tracts. The plot reports the average Area Under Curve (AUC) scores for ten tracts over 15 subjects. Expectedly, the AUC score increases as the number of examples increase.

We compared our proposed LAP-based supervised tract segmentation method against the automatic ROI-based segmentation, see Zhang et al. (2010), and the example-based multi-atlas approach with nearest neighbor strategy (hereafter denoted as NN_MULTI_ATLAS) of Yoo et al. (2015). The experiments are divided in two parts. In the first part, we segmented tracts with the three methods, i.e., ROI-based, NN_MULTI_ATLAS, LAP-based and measured the degree of voxel overlap with the ground truth, see Figure 4. In the second part, we performed a more in-depth analysis between the nearest neighbor strategy and the proposed LAP-based segmentation, ruling out confounds such as the different refinement strategy, the different embedding and different streamline distance function. In order to do that, we first show that the NN_MULTI_ATLAS method and our efficient nearest neighbor implementation (hereafter denoted as NN_DR_MAM, see section 3.2.1) provide very similar results, see **Figure 7**, despite the differences in the refinement step, embedding and streamline distance function. Then, we show the results of a ROC/AUC analysis comparing NN_DR_MAM and the proposed LAP-based segmentation, illustrating the advantage of LAP over nearest neighbor, see **Figure 8**, Table 2. Moreover, we provide additional results that enrich our work, such as visual examples of segmented tracts by the different methods, see Figures 5, 6, or the role of the number of examples for the quality of segmentation, see **Figure 9**. In the following, we provide all the necessary details and descriptions of the results and how we obtained them.

**Table 2:** Summary of the results: The average area under the Receiver operating characteristic (ROC) analysis between segmented tracts and ground truth tracts for the NN_DR_MAM and the proposed LAP.

**Method**		
**Tract**	**NN_DR_MAM**	**LAP**
CG.Left	0.81	0.90
CG.Right	0.81	0.88
IFOF.Left	0.70	0.86
IFOF.Right	0.68	0.80
UF.Left	0.69	0.84
UF.Right	0.70	0.83
CST.Left	0.63	0.79
CST.Right	0.66	0.75
ILF.Left	0.71	0.88
ILF.Right	0.67	0.84

*The table reports the area under curve (AUC) scores for each tract averaged over 15 subjects. For each tract the voxel information has considered for the ROC curve analysis and For each subject same threshold has chosen*.

### 5.1. Performance analysis

To quantitatively evaluate the proposed LAP-based supervised segmentation, we adopted two different evaluation metrics: (i) the dice similarity coefficient (DSC) (Dice, 1945) and (ii) the Receiver Operating Characteristic (ROC) curve (Brown and Davis, 2006) analysis. In the following, we will discuss these metrics in details.

#### 5.1.1. Dice similarity coefficient (DSC)

The common practice to evaluate tract segmentation methods is to compute the overlap, in terms of voxels, between the segmented tract and the ground truth tract (Jin et al., 2014). The degree of voxel overlap can be measured through the common dice similarity coefficient (DSC). In order to compute the DSC, we converted the segmented tract and the ground truth tract into the binary mask where 1 indicates that a voxel is crossed by a streamline of the tract and 0 otherwise. Given the segmented tract, $\hat{t}$, and the ground truth tract *t*

(6) $$\begin{array}{l} DSC=\frac{2\times \left(\left|v\left(\hat{t}\right)\cap v\left(t\right)\right|\right)}{\left|v\left(\hat{t}\right)|+|v\left(t\right)\right|} \end{array}$$

where *v*(*t*) is the set of voxels crossed by the streamlines of *t* and |*v*(*t*)| is the number of voxels of *v*(*t*). The DSC varies between 0 to 1 and higher values indicate better overlap.

#### 5.1.2. Receiver operating characteristic (ROC) curve

The analysis of the ROC curve is a standard measure for performance in the field of the medical image segmentation techniques (Southall et al., 2000). It considers all possible degrees of sensitivity/specificity of the segmentation method under evaluation. We adopted such analysis to compare the performance of our proposed LAP-based segmentation method with respect to the nearest neighbor method.

The ROC curve plots the sensitivity, i.e., the *true positive rate* (TPR), against the specificity, i.e., *false positive rate* (FPR) of the segmentation method at different thresholds. Here, the threshold refers to the number of streamlines to consider as segmented tract after ranking the streamlines that we obtain in the refinement step, where we combine the segmentations obtained from each single example in a single ranking, see section 22. In the proposed LAP-based method, we set such threshold as the median number of streamlines across the example tracts. In order to compute the ROC curve, here we span through all possible thresholds in the ranking. The TPR and FPR are computed by comparing the streamlines above threshold (*positives*) and below threshold (*negatives*) with the ground truth, combining four numbers: the true positives (TP), i.e., the number of voxels of positives that are also in the ground truth tract, the false positives (FP), i.e., the number of voxels of the positives not in the ground truth, the true negatives (TN), i.e., the number of voxels of negatives not in the ground truth and the false negatives (FN), i.e., the number of voxels of negatives also in the ground truth. Hence, the TPR and FPR are formulated as follows:

(7) $$\begin{array}{l} TPR=\frac{TP}{TP+FN} \end{array}$$

(8) $$\begin{array}{l} FPR=\frac{FP}{FP+TN} \end{array}$$

High values of TPR and (1-FPR) means good segmentation. Additionally, the ROC curve can be summarized in a single scalar value: the *Area under ROC curve* (AUC) score, where higher AUC indicates better segmentation.

### 5.2. ROI-based tract segmentation

For each test subject, the 10 tracts of interest were segmented based on their related pair of cortical ROIs, obtained from the atlas in MNI space available at http://lbam.med.jhmi.edu/cmrm/Data_Yajing/fiberMenu.htm, see Zhang et al. (2010). For each subject and tract, given their two ROIs, the segmented/estimated tract was obtained by keeping the streamlines of the tractogram that intersected them. The quality of the segmented tract, with respect to the ground truth, is reported in Figure 4, as DSC, see the red bars. The segmentation of a single tract took, approximately, a few seconds of computation on a modern desktop computer.

### 5.3. Tract segmentation with nearest neighbor (NN_MULTI_ATLAS method)

Following the NN_MULTI_ATLAS method of Yoo et al. (2015), we segmented each of the 10 tracts of interest for each subject of the test set using the example tracts from the 15 example subjects. First, all streamlines of all tractograms, from the example set and test set, were re-sampled to 32 points. Then, given an example tract of interest from the example set and the tractogram of a test subject, we computed the nearest streamlines of that example in the test tractogram, using the Mahalanobis distance. As in Yoo et al. (2015), the nearest neighbor computation was carried out with the trivial algorithm, i.e., by first computing all possible pairwise distances between the streamlines of the example tract and the test tractogram and then by keeping the one at minimum distance. We repeated such procedure for all 15 examples of the example set and pooled all the obtained segmented streamlines. Following Yoo et al. (2015), we excluded all streamlines distant more than 200 units (in Mahalanobis distance) from their corresponding example streamline and then ranked the remaining ones according to the number of times they were selected by different examples. As in Yoo et al. (2015), we defined the resulting segmented tract as the set of streamlines that were selected by at least half of the examples. The quality of the segmented tract, with respect to the ground truth, is reported in Figure 4, as DSC, see the blue bars.

The segmentation of a single tract took, approximately, from half an hour to more than two hours, depending on the size of the tracts. The segmentations were conducted on a modern desktop computer, specifically with an Intel Xeon processor, 16 Gb of RAM, 8 cores. The segmentations were computed in parallel, one example per core. Our implementation was in Python code, using the Free/OpenSource libraries NumPy, SciPy and DiPy^9^. The core part of the computation, i.e., the computation of all pairwise Mahalanobis distances, was implemented in C language, using scipy.spatial.cdist(). We could not use the GPU implementation of NN_MULTI_ATLAS mentioned in Yoo et al. (2015), because not provided by the authors.

### 5.4. Tract segmentation with LAP

As mentioned in section 3, our proposed LAP-based supervised tract segmentation method consists of two parts: (i) Tract correspondence from a single example, through LAPJV and (ii) merging of the correspondences from multiple examples through ranking. Here we discuss the choice of the parameters in each part.

**Figure 10:**
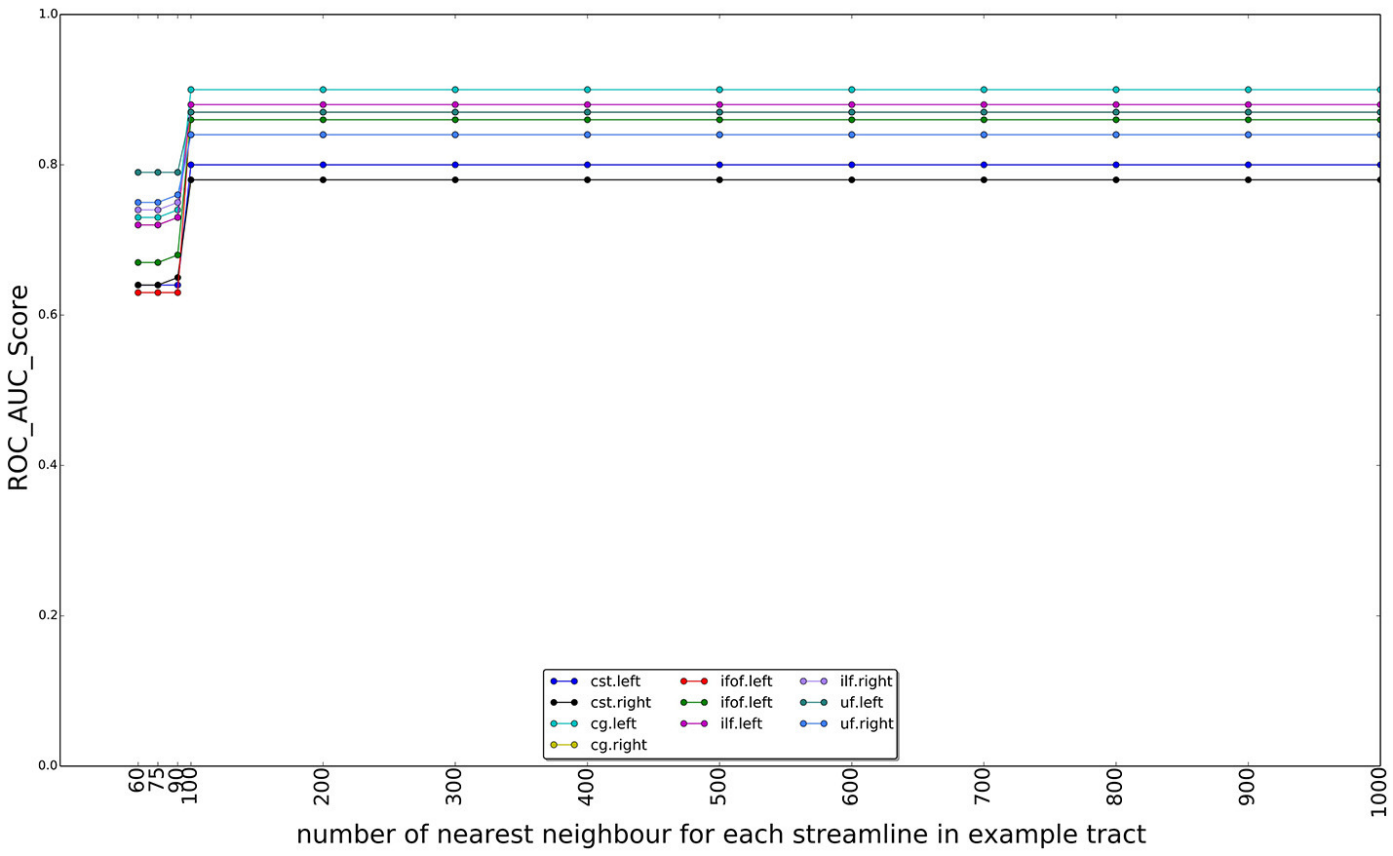
Change on the AUC score with the number of the nearest neighbors for each streamline in example tract used to compute the superset of each example, in order to sparsify the cost matrix of the LAP. The plot reports the AUC scores for ten tracts over 15 subjects as the number of nearest neighbors increases from 60 to 1,000. After 100 neighbors the results are stable, i.e., the target tract is always included in the superset.

As described in section 3.2.1, tract correspondence from a single example was obtained by solving the LAP between the streamline of the example and those of the target tractogram, with the rectangular LAPJV. Due to the computational cost of the algorithm, we could not directly apply it to the full cost matrix, computed between the streamlines of the example tract and the whole set of streamlines of the target tractogram. For this reason, the LAPJV algorithm was applied to a reduced cost matrix. The reduced cost matrix was computed by considering only a subset of the streamlines of the target tractogram *T*_*B*_, specifically the union of all 500 nearest neighbors of the streamlines in the example tract *t*_*A*_, *i*∈{1, …, *N*}. We expect such union set to be a superset of the ground truth tract *t*_*B*_ and we motivate the choice of 500 neighbors below, see section 3.2.1 and **Figure 10**. In this way, instead of computing a cost matrix of size (approximately) 10^2^×10^5^ and solve the corresponding LAP, we reduced the problem to a cost matrix of size approximately 10^2^×10^3^. In order to compute such superset, we used the dissimilarity representation (DR) and a *k*d-tree. According to Olivetti et al. (2012), 40 prototypes were selected from the whole brain tractogram, *T*_*B*_, using the SFF heuristic and the MAM distance function. With them, we computed the DR, i.e., a 40-dimensional vectorial representation, of each streamline in *T*_*B*_. Afterwards, we put such vectors in a *k*d-tree, using the implementation provided by scikit-learn^10^. In the same way, all streamlines in the example tract *t*_*A*_ were represented as vectors using the previous prototypes. For each of those vectors, we computed 500 nearest neighbors from the *k*d-tree of *T*_*B*_ and defined the superset of the ground truth *t*_*B*_ as their union. Then, in order to compute the reduced cost matrix, the MAM distance between the each streamline in the example tract *t*_*A*_ and those in the union set, were computed. Finally, the LAPJV algorithm was applied to the constructed cost matrix, in order to obtain the corresponding streamline from a single example (*t*_*A*_).

Afterwards, we proceeded to the second step, considering multiple examples, i.e., the same anatomical tract from the 15 example subjects. We repeated the segmentation procedure as above for each of the 15 examples, obtaining 15 segmented tracts. After computing the union of those 15 sets of streamlines, we ranked the streamlines according to the frequency in which they appeared in the segmentations, as described in section 3.2.2. The resulting segmentation of tract of ground truth tract *t*_*B*_, that we referred to as ${\hat{t}}_{B}$, was obtained by taking the first *m* streamlines in the ranking, where *m* is the median number of streamlines among the example tracts^11^.

We qualitatively evaluated our proposed segmentation method first with the DSC and then with the ROC curve analysis (see later). We measured the degree of overlap between the tract segmented by the LAP, ${\hat{t}}_{B}$ and the ground truth tract, *t*_*g*_, through the DSC, at the voxel-level. We computed the average and standard deviation of the mean DSC values for each ten tracts from the 15 subjects of the test group. The results are reported in Figure 4.

We implemented the proposed LAP-based approach with a combination of Python and Cython^12^ code, leveraging Numpy, SciPy, scikit-learn (for *k*-d tree) and Pymatgen (for LAPJV). We developed a parallel version of the proposed method in order to compute the segmentation of multiple subjects at the same time, across the available cores of the CPU. On average, on a modern desktop computer^13^, for each subject, the DR took approximately 30 seconds of computation time, whereas the *k*-d tree construction needed 20 seconds, for the whole brain tractogram. The correspondence with LAPJV required 10–30 seconds per tract, depending on the number of streamlines of the tract. In total, the segmentation of one tract from 15 examples required less than two minutes of computation. The software implementation of the proposed method is available under a Free/OpenSource license from here: https://github.com/FBK-NILab/LAP_tract_segmentation.

We further validated our proposed segmentation technique in a visual way. Figures 5, 6 presents tracts as segmented by our proposed segmentation method (LAP) and the corresponding ground truth (GT). For all the tracts considered in this experiments, we report the segmentations with highest DSC (first column) and lowest DSC (third column), together with the respective ground truths (second and fourth column). In the figure, for LAP, we indicate the correctly segmented streamlines (TP) in salmon color and the incorrect ones (FP) in green. For GT, we indicate again the TP of LAP in salmon color and the missed streamlines (FN) in yellow color.

### 5.5. LAP vs. NN: ROC/AUC analysis

We further investigated the differences between the proposed method, based on the LAP, and the one of Yoo et al. (2015) based on the nearest neighbor (NN) strategy. Here, our main aim is to make the most direct comparison between the two different principles used to find corresponding streamlines: LAP using a *joint* minimization of distances over all streamlines (imposed by the one-to-one constraint) and NN using instead a *greedy* distance minimization of each streamline, individually.

We noticed that the NN_MULTI_ATLAS method of Yoo et al. (2015) differs from the proposed one on multiple aspects: the embedding procedure (re-sampling to 32 points vs. dissimilarity representation), the streamline distance function (Mahalanobis vs. MAM), the refinement step (majority-vote ranking with two thresholds vs. nesting of two rankings and median threshold, see section 22) and the minimization function (NN vs. LAP). Since we are interested only in the last one of such differences, we wanted to avoid the effects of the other differences which could be confounds affecting the result. For this reason, we created a different NN implementation, based on the DR, *k*-d tree, MAM distance and the proposed refinement step, that we called NN_DR_MAM, and used instead of NN_MULTI_ATLAS. In section 3, we have already shown that with these new elements we can retrieve the nearest neighbor streamlines in a very short time (milliseconds), making this alternative implementation of NN very convenient from the computational point of view with respect to the one of Yoo et al. (2015). Despite all the differences introduced in our implementation of NN, we verified that the quality of segmentation of NN_DR_MAM did not substantially differ from NN_MULTI_ATLAS. In Figure 7, we report the DSC values obtained with the two implementations over all 150 segmented tracts. The interpolating line, obtained with a robust linear regression algorithm^14^, has a slope of 0.89, showing that NN_DR_MAM is equal or marginally superior to NN_MULTI_ATLAS.

In Table 2 we report the summary of the results of the ROC/AUC analysis of NN_DR_MAM vs. LAP, where the threshold changed during the sensitivity/specificity analysis was the number of streamline considered starting from the top of the ranking. In Figure 8, we report the detailed ROC curves for each tract, macro-averaged over the 15 test subjects.

### 5.6. LAP segmentation: number of examples and of neighbors

In this last part of the experiments, we investigated the effect of two important parameters on the proposed LAP segmentation: (i) the number of example tracts, i.e., 15 examples, and (ii) the number of nearest neighbors when building the superset for massively reducing the computational cost of LAP.

In Figure 9, we plotted the quality of the segmentation in terms of ROC/AUC score for each tract, averaged over all test subjects, when the number of example tracts varied from 1 to 15. Due to the variability of anatomy across subjects, having a larger set of examples is desirable for segmenting. Expectedly, all graphs show an increase in the score when the number of examples increases. The increase between 10 and 15 is modest, indicating that 15 examples are a meaningful example set size for segmenting.

In Figure 10, we show the effect of using different numbers of nearest neighbors of example tract when computing the superset on which the LAP is carried out. The graphs show the ROC/AUC score for each tract of interest, averaged over the test subjects when the number of neighbors span from a few tens to 1000. A small number of neighbors for each example streamline result in a small superset. It is expected that, if such superset is too small, then it might not contain all the streamlines of the actual target tract, i.e., the ground truth, preventing the proposed LAP method to get good quality of segmentation. In other words, a too small superset acts as an artificial upper bound on the score of LAP. Nevertheless, all the graphs show that, when the number of neighbors is above a 200, such issue completely disappears, for all tracts. Notice that, in the experiments, we always used 500 neighbors, a value which safely larger and, thus, justified.

## 6. Discussion

In this section, we discuss the results obtained in section 5 and show their impact on our claims.

We performed a set of experiments where the proposed LAP-based segmentation method is compared against two alternative methods: (i) the commonly used automatic ROI-based segmentation (Zhang et al., 2010) and (ii) a recent example-based multi-atlas method based on the idea of correspondence implemented with the nearest neighbor algorithm (NN_MULTI_ATLAS) (Yoo et al., 2015). The quality of segmentations of 10 tracts for 15 test subjects, obtained by each of the 3 methods, was scored according to their voxel-overlap (as DSC) with the ground truth, obtained through WMQL (Wassermann et al., 2013). Figure 4 reports the result of average DSC for 10 tracts, averaged over 15 test subjects. For each tract, three bar plots are reported, one for each segmentation method, along with the standard deviation of mean. The bar-plots show that proposed LAP-based method is able to segment tracts much better than the automatic ROI-based segmentation and remarkably better than example-based segmentation with the NN strategy. Additionally, notice that our results independently reproduce the result reported in Yoo et al. (2015), on a different dataset, where the multi-atlas NN method outperformed the ROI-based segmentation.

Across all tracts, our method showed strongly improved results, in terms of voxel overlap, with respect to ROI-based segmentation, usually doubling (or more) its score, i.e., from 0.15 to 0.40 vs. 0.45 to 0.75. The result from the ROI-based segmentation approach is indeed quite modest with respect to the other methods, as already seen in Yoo et al. (2015). This could be due to the limitations of registration, occurring when aligning the test subject to the cortical atlas.

Figure 4 showed that LAP-based segmentation is a substantial improvement over the NN-based method of Yoo et al. (2015). Nevertheless, we conducted a further analysis to make a detailed and more general comparison between the greedy optimization of the NN strategy, which defines the corresponding streamlines as those with minimum distance, and the proposed solution based on the LAP, which *jointly* minimizes the distances among all example streamlines and the target tractogram, introducing a one-to-one constraint. Our claim is that the segmentation with the NN strategy may fail in some cases, like when there is still a local systematic displacement between homologous anatomical structures, after the tractograms of the example and target subjects are registered. In such cases, the tract segmented with NN will be under estimated, see Figure 1. Differently, the one-to-one constraint of LAP should force the correspondence to correctly follow such displacement.

In order to conduct a careful analysis without confounds, we implemented an alternative version of the multi-example NN segmentation algorithm of Yoo et al. (2015), in order to match all the aspects of the proposed LAP-based segmentation method, see section 5.5. We supported such argument by showing that our implementation, called NN_DR_MAM, provides equivalent results to that of Yoo et al. (2015), called NN_MULTI_ATLAS, see Figure 4. Then, we conducted a ROC/AUC analysis between NN_DR_MAM and our LAP-based segmentation. In Figure 8 we presented the ROC curves for each tract of interest, averaged over the 15 test subjects. Those curves are summarized in Table 2, where the ROC/AUC scores are reported. The LAP-based segmentation always obtains high ROC/AUC scores, i.e., 0.75–0.90, always remarkably higher than the ones of NN, i.e., 0.63–0.81, proving our claim. Notice that this result is even more insightful than that of Figure 4, because the ROC/AUC analysis is independent from the arbitrary choice of the number of streamlines for the target tract that we defined as the median value of the size of the example tracts. In other words, this result is independent from the choice of the threshold that we introduced with the proposed method.

Another important aspect of the proposed method is its computational cost. In the experiments, we have shown that the time required to segment one tract from 15 examples may be even more than two hours of computation, when using the NN method of Yoo et al. (2015). Instead, the proposed LAP-based segmentation requires only a couple of minutes of computation, obtaining also a higher quality of the results. Nevertheless, with the efficient computational tools of the dissimilarity representation and *k*-d tree adopted for the proposed method, we also implemented an alternative version of the multi-example NN segmentation method, called NN_DR_MAM. We have shown that it requires even less time than the LAP-based segmentation and obtains comparable results to those of Yoo et al. (2015). Our fast implementation of NN-based segmentation from multiple examples is comparable to that of Labra et al. (2017) in terms of speed-up.

Despite all the positive results, there is notable variability in the quality of segmentations of all methods, including the proposed LAP-based one. In Figures 5, 6 we show 20 examples of segmented tracts obtained with the proposed LAP-based method, together with the respective ground truth (GT) obtained with WMQL. There, we show 2 examples of segmentations for each of the tracts of interest. These two examples are the one where the LAP-based method obtained the highest DSC score (column A) and the one with lowest DSC score (column B). The salmon-colored streamlines are the correctly segmented ones (TP). The green streamlines are the incorrectly segmented ones (FP) and the yellow streamlines are the missed ones (FN). In column A (highest DSC), for all the 10 tracts considered in this study, the large majority of streamlines are correctly segmented. Differently, in column B (lowest DSC), many tracts are poorly segmented. This fact occurs for two reasons: first, despite remarkable progress with respect to previous automatic segmentation methods, the proposed LAP-based segmentation cannot be considered as a final answer. The problem of automatic tract segmentation, even with the support of multiple examples, still have substantial room for future improvement. Secondly, the quality of the ground truth, both for examples and test tracts, should be improved. As it is clearly visible in Figures 5, 6, column B, some of the ground truth tracts are questionable, e.g., CST Right and ILF left, and could be easily challenged by an expert neuroanatomist. If the ground truth is poor, we cannot expect example-based segmentation methods to succeed. The common use of WMQL for defining the ground truth is appealing because of its low cost: like in our case, obtaining 300 high-quality segmentations from expert neuroanatomists would be vastly beyond reach from a single research group. At the same time, the limited quality of some of the tracts limits the generalization ability of example-based methods. This limitation is also a call to our community to collaboratively share segmented tracts by experts, in order to reach sufficient numbers to enable creation and assessment of higher-quality automatic methods.

One important question is about the size of the example set, that we set to 15 examples in our study. Given the variability of white matter anatomy in the healthy population, it is important to understand what is the trade-off between the cost of acquiring more examples and the gain in quality of segmentation. We analyzed this aspect and reported the results in Figure 9, where we observed the expected increase in ROC/AUC score when increasing the number of examples used by the proposed LAP-based segmentation method. This increase confirms what already has been observed in Yoo et al. (2015 see Figure 11 there) for the case of NN-based segmentation and, in comparison to that study, in Garyfallidis et al. (in press see Tables 1, 2 there, in particular the Jaccard index^15^), where a variation of the NN from a single example is presented. For our LAP-based segmentation, we also observed moderate improvement of the quality of segmentation when using more than 10 examples, justifying the choice of 15 presented in this paper. Of course, such result may be limited by the quality of the ground truth tracts. With more homogeneous ground truth tracts, we might expect a steeper curve than the one in Figure 9, which would shift the desirable size of the set of examples to a higher value.

Although our proposed LAP-based tract segmentation method showed very positive experimental results, there is a critical parameter: the threshold that defines the number of streamlines for the segmented tract. In this work, we defined such threshold as the median value among the sizes of the example tracts. The median is a simple sensible choice because it is the number that deviates less from what has been observed in the example set. Moreover, it is more robust to outliers than the sample mean value. Nevertheless, such threshold is based only on example data and ignores the specificity of the test subject. This current limitation reveals an implicit assumption of our method, as well as the ones based on NN, i.e., that the number of streamlines of the tract of interest across all subjects should not change much. Besides the unavoidable subject variability in the white matter anatomy, the main implication of this assumption is that example and target tractograms should have a similar number of streamlines. Frequently, this is the case only when all tractograms are generated with the same tracking algorithm and same parameters. Different tracking algorithms, or even different parameter values, usually result in significant differences in a number of streamlines of the tractogram. As a consequence, both NN and LAP algorithms should not be used on non homogeneous (or inhomogeneous) sets of tractograms, at the current state.

The one-to-one constraint introduced by the LAP-based segmentation gives a clear advantage over NN-based segmentation but, sometimes, it can be seen as a limitation. Even in the case of homogeneous tractograms, the number of streamlines of a given tract of interest may substantially vary across subjects. In such case, each LAP will find the exact same number of streamlines between example and target tractogram, which may be sub-optimal. In future, we plan to refine and partly relax this constraint to find a more convenient compromise between the who extremes of NN and LAP.

## 7. Conclusions and future work

In this work, we present a novel method for supervised tract segmentation based on the idea of streamlines correspondence across subjects as a set of linear assignment problems (LAPs). Our proposed segmentation method is able to segment a given tract of interest in the tractogram of a new subject using a set of example tracts from other subjects, as prior information. The results of multiple experiments show that the proposed method provides a very large improvement over ROI-based segmentation and a remarkable improvement over nearest-neighbor (NN) segmentation. The time required to segment a tract of interest is just a few minutes and we provide a Free/OpenSource implementation of the proposed method.

The proposed method has some limitations, on which we plan to work in near future. First, we plan to improve the reliability of the results presented in this work by adding higher-quality tracts segmented by expert neuroanatomists, thus extending the current large dataset of 300 tracts used in our experiments. Secondly, we plan to address the limitation of defining the size of the target tract just from the examples, i.e., with the median value, that currently does not adapt to the target tractogram. To conclude, we plan to address automatic segmentation between tractograms generated from different tracking algorithms and with different parameters values. This last challenge should have an important impact on the adoption of supervised automatic segmentation systems, because we cannot expect to always have segmented tracts from homogeneous tractograms during deployment. In such cases, practitioners should provide homogeneous segmented examples before being able to use the supervised segmentation systems at their best.

## Author contributions

NS: conceived the ideas, designed the experiments, conducted the experiments, and draft the manuscript. EO: contributed to the ideas and design of the experiments and wrote the manuscript. PA: contributed to the ideas and to the manuscript.

### Conflict of interest statement

The authors declare that the research was conducted in the absence of any commercial or financial relationships that could be construed as a potential conflict of interest.

## Footnotes

^1^Two sets of streamlines, in our case.

^2^Usually, *M* ≈ 10^5^–10^6^.

^3^Notice that a streamline is not a vector but a sequence of points in 3D space and that different streamlines are sequences of different length, in general.

^4^We measured the timing using the *k*-d tree implementation sklearn.neighbors.KDTree provided by the Python package Scikit-learn, see http://scikit-learn.org, and the tractograms in our experiments represented as vectors through the DR.

^5^The DR of *t*_*A*_ is obtained using the prototypes of *T*_*B*_.

^6^http://nipy.org/dipy.

^7^https://github.com/demianw/tract_querier.

^8^http://fsl.fmrib.ox.ac.uk/.

^9^http://nipy.org/dipy.

^10^http://scikit-learn.org.

^11^In case of ROC/AUC computation, such number varied, in order to explore sensitivity and specificity of the method.

^12^http://cython.org/.

^13^8 cores CPU, Intel Xeon 3.50 GHz, 16 Gb RAM.

^14^The Huber regression algorithm.

^15^Given the much larger size of the segmented tract with respect to the ground truth, the main value to consider against other segmentation methods is the Jaccard index.
